# Sex Differences in Characteristics of Patients with Infective Endocarditis: A Multicenter Study

**DOI:** 10.3390/jcm11123514

**Published:** 2022-06-18

**Authors:** Ruchi Bhandari, Shabnam Tiwari, Talia Alexander, Frank H. Annie, Umar Kaleem, Affan Irfan, Sudarshan Balla, R. Constance Wiener, Chris Cook, Aravinda Nanjundappa, Mark Bates, Ellen Thompson, Gordon S. Smith, Judith Feinberg, Melanie A. Fisher

**Affiliations:** 1 Department of Epidemiology and Biostatistics, School of Public Health, West Virginia University, Robert C Byrd Health Sciences Center North, 64 Medical Center Drive, Morgantown, WV 26506, USA; st0090@mix.wvu.edu (S.T.); ter0012@mix.wvu.edu (T.A.); gordon.smith@hsc.wvu.edu (G.S.S.); 2 Health Education and Research Institute, Charleston Area Medical Center, Charleston, WV 25304, USA; frank.h.annie@camc.org; 3 Joan C. Edwards School of Medicine, Marshall University, Huntington, WV 25755, USA; kaleemu@marshall.edu (U.K.); irfana@marshall.edu (A.I.); ethompson@marshall.edu (E.T.); 4 School of Medicine, West Virginia University, Morgantown, WV 26506, USA; sudarshan.balla@wvumedicine.org (S.B.); chris.cook@wvumedicine.org (C.C.); judith.feinberg@hsc.wvu.edu (J.F.); mfisher@hsc.wvu.edu (M.A.F.); 5 School of Dentistry, West Virginia University, Morgantown, WV 26506, USA; rwiener2@hsc.wvu.edu; 6 Department of Cardiovascular Medicine, Charleston Area Medical Center, Charleston, WV 25304, USA; ananjundappa@hsc.wvu.edu (A.N.); mark.bates@camc.org (M.B.)

**Keywords:** infective endocarditis, West Virginia, sex, male-female differences, electronic medical records

## Abstract

Infectious diseases like infective endocarditis (IE) may manifest or progress differently between sexes. This study sought to identify the differences in demographic and clinical characteristics among male and female patients with IE. Data were obtained from a newly developed registry comprising all adult patients with first IE admission at the four major tertiary cardiovascular centers in West Virginia, USA during 2014–2018. Patient characteristics were compared between males and females using Chi-square test, Fisher’s exact test, and Wilcoxon rank-sum test. A secondary analysis was restricted to IE patients with drug use only. Among 780 unique patients (390 males, 390 females), significantly more women (a) were younger than males (median age 34.9 vs. 41.4, *p* < 0.001); (b) reported drug use (77.7% vs. 64.1%, *p* < 0.001); (c) had tricuspid valve endocarditis (46.4% vs. 30.8%, *p* < 0.001); and (d) were discharged against medical advice (20% vs. 9.5%, *p* < 0.001). These differences persisted even within the subgroup of patients with drug use-associated IE. In a state with one of the highest incidences of drug use and overdose deaths, the significantly higher incident IE cases in younger women and higher proportion of women leaving treatment against medical advice are striking. Differential characteristics between male and female patients are important to inform strategies for specialized treatment and care.

## 1. Introduction

Infective endocarditis (IE) is a potentially lethal disease that carries an in-hospital mortality approximating 20% [1,2]. Further, IE can lead to several cardiovascular complications, with heart failure occurring in up to 40% of patients [3]. Outcomes are worse for IE patients with underlying cardiovascular disease (CVD), especially those with valvular sclerosis [4]. Earlier estimates of IE incidence in the United States were 10–15 per 100,000 population [5], but the number of cases has increased sharply in recent years, predominantly attributable to IE acquired through injection drug use [6].

Many infections present and manifest differently in male and female patients. The physiological and anatomical differences in males and females may result in differences in the exposure, transmission, and control of a pathogen [7]. The immune system is also affected by sex [8]. Despite sex differences influencing the response to infectious diseases, they are largely overlooked [9]. Indeed, IE reportedly occurs more frequently in men, with a male-female ratio ranging between 2:1 and 9:1 [10,11,12,13]. Beyond this, however, sex differences are not adequately defined for causative agents, pre-exiting conditions, management, and outcomes among IE patients.

The few existing studies on sex differences in IE yielded contradictory findings [10,13,14,15,16]. In two prospective studies of patients with IE, results showed sex differences in univariate analysis; however, multivariable analyses demonstrated that underlying comorbidities or preoperative risk factors, and not sex differences, were the reasons for the observed differences in the clinical outcomes between male and female patients with IE [13,17]. Sex differences were also not evident in treatment strategies, in-hospital outcomes, or mortality in patients with IE [15,16]. In contrast, a recent retrospective study reported significant differences between sexes in infective organisms found in IE [14]. Female sex was found to be independently associated with higher mortality among patients with IE [10,14]. Additionally, among patients with native valve IE, men are more likely to have aortic valve involvement while females are more likely to have mitral valve involvement [17].

Sex can affect the risk of presentation of several health conditions, including cardiovascular diseases (CVD). Females have a lower risk of CVD before menopause, which, from animal model studies, is attributed to the potential protective roles of estrogen against endothelial damage [18,19]. However, this protective effect is abated by female-specific CVD risks such as hormonal contraceptives use, pregnancy related complications, and menopausal transition, all of which can increase CVD risk [20]. Importantly, the higher rate of CVD comorbidities in post-menopausal women could increase their risk of a severe presentation of IE with unfavorable outcomes. Comorbidities could also contribute to the observed sex differences in the management and outcomes of IE [13]. Conversely, physiological differences and manifestation of IE could further amplify its morbidity and mortality in female patients. Moreover, patients with more severe presentation of IE may not be eligible for invasive therapeutic options, given inherent morbidity/mortality risk associated with the intervention.

A richer understanding of potential sex differences in IE would benefit approaches to prevention, treatment, and management of IE. In this study, we assessed demographic and clinical characteristics by sex for patients with IE and their substance use behaviors, capturing most of the cases in an Appalachian state (West Virginia, WV) with one of the highest incidences of drug use and overdose deaths in the United States of America.

## 2. Methods

This study utilized data from a recent database developed from a comprehensive multisite chart review of electronic medical records of all adult patients ≥18 and <90 years of age with IE admitted at the four major tertiary cardiovascular centers in WV between 1 January 2014 and 31 December 2018. The hospitals in the study are the only tertiary centers in the state with the capability to perform heart surgery, and patients are referred to these centers from across the entire state. The team of cardiologists, infectious disease specialists, and other providers that worked on this project have a consensus based on discussions with colleagues that 90–95% of the IE cases in the state are likely to be treated in these four centers, and therefore the database is as representative of IE cases in the entire state as is possible.

Only the first admission was considered for each patient. Patients were identified by IE-associated ICD 10 codes (B376, I33.0, I33.9, I34.0, I34.8, I35.0, I35.1, I35.2, I35.8, I35.9, I36.0, I36.8, I37.0, I37.8, I38, I39) included in their electronic medical records. Manual chart reviews were performed for confirmed IE diagnosis. Study data were collected and managed using REDCap electronic data capture tools hosted at West Virginia University [21]. West Virginia University served as the Institutional Review Board of oversight and approved the study (IRB protocol number: 1811373348).

Data on race (white vs. other/mixed), age (continuous), smoking status (current smoker, ex-smoker, non-smoker), alcohol use (current alcohol use, ex-alcohol use and no alcohol use), drug use before hospital admission, discharge status, including discharged against medical advice (AMA) and death, comorbidities, psychiatric disorders, affected valve, and causative organisms are presented by sex (male/female). The microbiology labs in all centers used the MALDI-TOF mass spec identifications as the primary method for identification of yeast and bacteria.

We present categorical variables as counts and percentages. Chi-square test or Fisher’s exact test when expected cell count was <5 were used to compare between groups with two categories. Continuous variables are presented as median and interquartile range (IQR). Medians were compared using the nonparametric Wilcoxon rank-sum test. Statistical analyses were conducted using SPSS version 27. Statistical significance was accepted at *p* < 0.05. P-values were highlighted for statistical significance in the tables after adjustments were made using Bonferroni correction wherever multiple tests were conducted. In addition, we conducted a secondary analysis among the subset of IE patients with drug use given the potential differences in characteristics associated with drug use.

## 3. Results

In a medical chart review of electronic health records covering the four largest university-affiliated hospitals in WV, we identified 780 unique patients with IE across 5 years. Incident IE cases were equal by sex, with 390 males and 390 females. The incidence of IE increased during the study period (2014–2018), but the increase was similar among males and females (Table 1).

**Figure 1:**
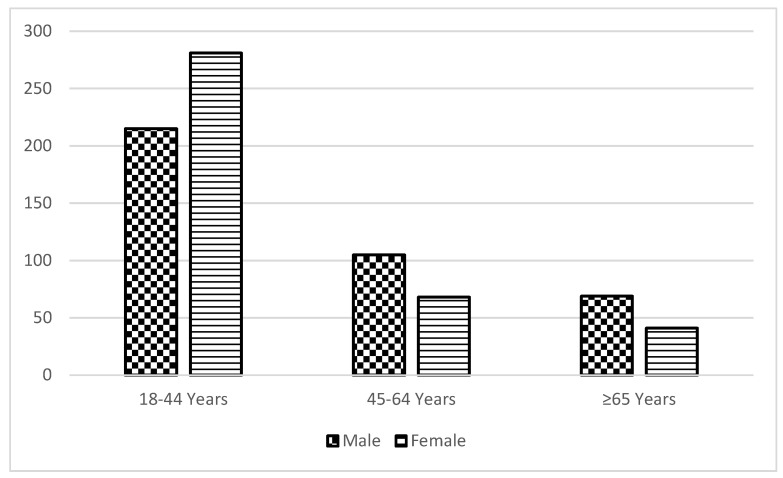
Number of male and female patients with infective endocarditis in West Virginia by age groups: 2014–2018.

Some demographic and clinical characteristics differed significantly by sex for patients with IE (Table 1). Female patients were significantly younger overall than males (median age 34.9 vs. 41.4, *p* < 0.001). Compared with males, a higher proportion of females were in the younger (18–44 years) age group (72.1% vs. 55.3%, *p* < 0.001) (Figure 1); were current smokers (71.8% vs. 56.2%, *p* < 0.001); had used drugs before hospital admission (77.7% vs. 64.1%, *p* < 0.001). There was no statistically significant difference between sexes over the years with respect to current alcohol use. However, we did see an increasing proportion of female patients with IE who were current smokers from 2014 to 2018 (data not shown).

Further, as presented in Table 2, a significantly higher proportion of women had some forms of psychiatric disorders, specifically, depression (27.2% vs. 14.4%, *p* < 0.001) and bipolar disorder (8.2% vs. 2.3%, *p* < 0.001). There was no significant difference between males and females concerning substance use disorder. Similarly, significantly more women had two or more psychiatric disorders (29.0% vs. 17.7%, *p* < 0.001). However, more men had comorbidities other than psychiatric disorders, such as coronary artery disease (15.9% vs. 8.5%, *p* = 0.001) and peripheral vascular disease (10% vs. 4.6%, *p* = 0.004). We did not find statistically significant difference between sexes with respect to comorbidities when analyzed separately by years (data not shown).

Male and female patients also differed in the clinical characteristics of IE. A significantly larger proportion of females compared to males had tricuspid valve endocarditis (53.8% vs. 36.4%, *p* < 0.001); more men had aortic valve endocarditis (35.6% vs. 15.4%, *p* < 0.001). A significantly larger proportion of men compared to women had IE caused by *Streptococcus*, including Viridans Streptococci (16.4% vs. 8.0%, *p* < 0.001) and *Enterococcus* species (10.8% vs. 4.6%, *p* = 0.001).

Hospital discharges also differed by sex. Significantly more men than women were discharged alive after treatment completion (80.3% vs. 71%, *p* = 0.003). Strikingly, the percentage of women discharged against medical advice (AMA) was twice that of men (20% vs. 9.5%, *p* < 0.001). When stratified by age (<50 vs. >=50) as a proxy for postmenopausal age, we found statistically significant difference in mortality among older women compared with younger women; but this difference did not exist among men (data not shown).

Table 3 shows the results among the subgroup of patients with drug use-associated IE, which were consistent with the characteristics of the overall sample. Female patients with drug use-associated IE were significantly younger overall than males (median age 35.7 vs. 32.2, *p* < 0.001). Significantly more women had two or more psychiatric disorders (34.6% vs. 25.6%, *p* < 0.039), with more depression and bipolar disorder. A significantly larger proportion of females compared to males had tricuspid valve endocarditis (65.7% vs. 44.8%, *p* < 0.001); more men had aortic valve endocarditis (29.6% vs. 12.2%, *p* < 0.001). Lastly, a significantly higher proportion of women were discharged against medical advice (24.8% vs. 14%, *p* = 0.002). In this sub-population, there were no statistically significant differences between men and women in MRSA-associated IE and substance use disorder, unlike the overall patients with IE. Over 70% of the patients with drug use had documentation in their charts that they injected drugs (data not shown).

## 4. Discussion

In this large retrospective study incorporating review of electronic medical records from the four largest university-affiliated hospitals in WV during 2014–2018, we found an equal incidence of IE among males and females, but several statistically significant sex-related differences in demographic and clinical characteristics. Female patients with IE were much younger overall, current smokers, had drug use—particularly injection drug use—before hospital admission, used opioids and polydrugs, were on medication for opioid use disorder before hospital admission, and had higher burden of psychiatric disorders, including, depression, anxiety, and bipolar disorder. However, when the analysis was restricted to patients with drug use-associated IE only, men and women were not significantly different in the diagnosis of substance use disorder, suggesting that the disorder was largely linked to drug use.

In this study, we observed that the incidence of hospital visits for IE grew annually across the study period for both males and females. This is consistent with a significant increase in the incidence of IE in the United States (US) [22]. While most studies reported a male predominance of IE [10,14,15,22,23,24], a few noted a higher proportion of females with IE [25,26]. The incidence of IE was similar in both sexes in our study. Data from the United States Census Bureau show that women make up about equal proportion (49.5%) of the state population and the proportion of male to female population has remained constant during the study period (2014–2018) [27]. 

Findings on the average age of male and female patients with IE have been contradictory. Several studies showed that male patients were significantly older than female patients [15,28]; others concluded the opposite [10,14]. Notably, prior work also indicated significant increases in IE rates with increasing age in both sexes [23,29]. In our study, we found a significantly lower average age for females with IE compared with males; the incidence of IE was also higher in younger females (18–44 years). Our results corroborate those of a study utilizing data from the Health Care and Utilization Project National Inpatient Sample between 2000 and 2013, which found a trend of injection drug use-associated IE hospitalizations shifting toward younger white females [30]. Estimates from a household interview survey of the US civilian population demonstrate that over 42% of the population, aged 18–34 years, reported non-medical use of medication or psychoactive substance in the past month compared to 8% of the people aged 35 years or older [31]. Findings demonstrate the higher prevalence of drug use in the younger population, the prevalent opioid epidemic in the geographic region, and injection drug use-related IE being the predominant etiology.

There is an association between smoking, non-medical drug use, and IE [32]. In our study, while a high proportion of both men and women with IE were current smokers, significantly more women than men were current smokers. WV data from the Behavioral Risk Factor Surveillance System (BRFSS) for the years 2016–2018 show no significant gender difference in the prevalence of current cigarette smoking: men—25.7%, 25.1%, 25.8%, and women—24.8%, 26.9%, 23.9% for the years 2018–2016, respectively—unlike the data from our study [33]. Compared to the BRFSS data, the proportion of smokers among our patient population was much higher (2.2 times higher in males and 2.8 times higher among female patients with IE). The prevalence of heavy drinking in BRFSS was significantly higher among men than among women in WV: with men having prevalences of 6.5%, 4.5%, and 4.6%, and women having prevalences of 2.1%, 2.7%, and 2.5% for the same years. Our data also show a significantly higher proportion of male patients with IE who had current alcohol use.

While findings from our study demonstrate significantly more female patients with tricuspid valve endocarditis, other studies found either equal involvement of the tricuspid valve among both males and females or more among male patients [17,34]. The tricuspid valve is commonly affected among the IE patients with injection drug use [24], and we observed a higher proportion of female patients with drug use in our study. Strikingly, unlike our results, other studies found a higher proportion of female patients who were diagnosed with mitral valve endocarditis [11,13,17,35]. Our observation of higher involvement of aortic valve among male patients with IE corroborate findings from other studies [11,17,36].

Interestingly, we also detected differences by sex in the causal organisms. A large cohort study demonstrated the role of host or pathogen-specific characteristics and association with poor outcomes in patients with IE [37]. Consistent with prior studies [38,39], S*taphylococcus* was the leading causative microorganism of IE in our study. However, MRSA organisms were detected in almost half (47.3%) of female patients with IE, which was significantly higher when compared with male patients. A recent study in patients with IE in Egypt showed that more males, compared with females, had Staphylococcal organisms, though the difference was not statistically significant [15]. Our study found that more men compared with women had other *Streptococcus* and *Enterococcus* species. However, we did not collect data on the bacteria classified as “other streptococci”. In our study, the difference in MRSA-associated IE ceased to exist when restricted to drug use-associated IE, indicating an association between drug use and MRSA infection.

Discharges AMA are associated with poor prognosis, including higher mortality and readmissions [40,41]. Yet, it is common for patients with IE to leave the hospital AMA, especially among people who inject drugs [40,42]. Our analysis showed females are more likely to leave AMA compared to males. These findings are different from many studies where the general trend is higher proportion of men being discharged AMA [40,43,44,45]. There are several possible reasons for discharge AMA, especially for women, which may include childcare, craving for drugs, poor pain control, opioid withdrawal, or dissatisfaction within the hospital setting. We did not observe a difference between in-hospital mortality in male and female patients, similar to another large prospective French study [11]. However, studies have found conflicting results, with higher in-hospital mortality in women [13,35] or men [46]. Since female patients in our study had a high rate of discharge AMA, a prospective cohort study following the patients who were discharged AMA is critical to understanding the reasons for these outcomes.

This study has some limitations. A limitation of the retrospective design is the lack of long-term follow-up data, especially for patient outcomes after discharge from hospital. Additionally, because WV’s population is primarily white [47], the results may not be generalizable to IE patients of other racial groups. While some IE patients in the state may not have been treated in our referral hospitals, the increase in female cases is unlikely be a result of differing referral patterns, as there are no other instate tertiary care hospitals available and most IE cases would not be able to afford out-of-state treatment.

The strengths of our study include data from multiple large tertiary care institutions that are geographically dispersed in the state of WV. We conducted a thorough medical record review of every patient, reviewing the information from several different sources within the record, such as hospital narratives, history and physical notes, operative notes, consultation notes, screening and test results, and discharge summary. Therefore, we were able to overcome the problem of missing information that generally arises from data retrieved only on the basis of ICD-CM codes. In addition, the availability of data in medical charts offered a low-cost option for us to develop the database that we used for this study. We adequately identified and operationalized the study variables, and trained and monitored the data abstractors. We used standardized abstraction form through REDCap that provided a centralized data storage location and reduced input and data transcription error. We had access to granular data for both male and female patients with IE, including the microbiologic etiology, type of IE (right versus left-sided), and comorbidities.

## 5. Conclusions

The significantly higher incidence of IE in younger women and higher proportion of women leaving treatment against medical advice are distinct features in a state with one of the highest incidences of drug use and overdose deaths. These differential characteristics between male and female patients suggest that younger females with injection drug use are more susceptible to developing IE than similarly aged males. More clinical and social-behavioral research is needed to explain the possible reasons for this discrepancy between younger males and females. Sex differences may help inform strategies for specialized treatment and care, and guide future research to better understand risk differences among male and female patients with IE.

## Figures and Tables

**Table 1:** Characteristics of male and female patients with IE* in WV: 2014–2018.

	Male		Female		*p*-Value
	N = 390	50.0%	N = 390	50.0%	
	N	%	N	%	
**Year**					0.543
2014	28	7.2	22	5.6	
2015	45	11.5	34	8.7	
2016	66	16.9	76	19.5	
2017	115	29.5	116	29.7	
2018	136	34.9	142	36.4	
**Race**					0.962
White/Caucasian	372	95.4	364	93.3	
Other/Mixed	10	2.6	10	2.6	
Missing	8	2.1	16	4.1	
**Smoking status**					**<0.001**
Current smoker	219	56.2	280	71.8	
Ex-smoker	80	20.5	32	8.2	
Non-smoker	55	14.1	43	11.0	
Missing	36	9.2	35	9.0	
**Alcohol use**					**<0.001**
Current alcohol use	90	23.1	65	16.7	
Prior alcohol use	54	13.8	14	3.6	
No alcohol use	187	47.9	221	56.7	
Missing	59	15.1	90	23.1	
**Drug use**					**<0.001**
Yes	250	64.1	303	77.7	
No	132	33.8	82	21.0	
Missing	8	2.1	5	1.3	
**Age**	**Median**	IQR**	**Median**	IQR**	**<0.001**
	41.42	26.87	34.94	19.02	

IE*: infective endocarditis; IQR**: inter-quartile range.

**Table 2:** Clinical features of patients with IE* stratified by sex.

	Male		Female		*p*-Value
	N = 390	50.0%	N = 390	50.0%	
	N	%	N	%	
**Causative organisms**					
MRSA^†^	148	37.9	185	47.4	**0.007**
MSSA^††^	91	23.3	112	28.7	0.087
Other	41	10.5	34	8.7	**<0.01**
Other Streptococci species	43	11.0	26	6.7	**0.032**
Enterococcus species	42	10.8	18	4.6	**0.001**
Candida species	22	5.6	26	6.7	0.551
Serratia species	19	4.9	23	5.9	0.526
Culture negative	16	4.1	16	4.1	1.000
Viridans Streptococci	21	5.4	5	1.3	**0.001**
Klebsiella species	9	2.3	7	1.8	0.613
**Comorbidities**					
Psychiatric disorders	142	36.4	178	45.6	**0.009**
Hypertension	132	33.8	98	25.1	**0.008**
Type 2 Diabetes	65	16.7	58	14.9	0.492
Coronary Artery Disease	62	15.9	33	8.5	**0.001**
Chronic lung disease	45	11.5	44	11.3	0.910
Hyperlipidemia	53	13.6	32	8.2	**0.016**
Acute kidney injury	36	9.2	41	10.5	0.548
Chronic kidney disease	45	11.5	25	6.4	**0.012**
Stroke	30	7.7	31	7.9	0.894
Peripheral vascular disease	39	10.0	18	4.6	**0.004**
Metastatic infections	15	3.8	24	6.2	0.139
**Number of comorbidities**					0.619
0	91	23.3	100	25.6	
1	135	34.6	138	35.4	
2	67	17.2	70	17.9	
3 or more	97	24.9	82	21.0	
**Psychiatric disorders**					
Substance Use disorder	111	28.5	122	31.3	0.389
Depression	56	14.4	106	27.2	**<0.001**
Anxiety	58	14.9	83	21.3	0.020
Bipolar disorder	9	2.3	32	8.2	**<0.001**
Post Traumatic Stress Disorder	12	3.1	24	6.2	0.041
**Number of psychiatric disorders**					**0.001**
1	72	18.5	63	16.2	
2	38	9.7	51	13.1	
3 or more	31	7.9	62	15.9	
0 or missing	249	63.8	214	54.9	
**Valve**					
Tricuspid	142	36.4	210	53.8	**<0.001**
Mitral	111	28.5	124	31.8	0.31
Aortic	139	35.6	60	15.4	**<0.001**
Pulmonic	10	2.6	13	3.3	0.525
**Discharge status**					
Discharge alive	313	80.3	277	71.0	**0.003**
Death	40	10.3	35	9.0	0.544
Against Medical Advice	37	9.5	78	20.0	**<0.001**

IE*: infective endocarditis; MRSA^†^: staphylococcus aureus, methicillin resistant; MSSA^††^: staphylococcus aureus, methicillin.

**Table 3:** Clinical features of patients with drug use-associated IE* stratified by sex.

	Male		Female		*p*-Value
	N = 250		N = 303		
	N	%	N	%	
**Smoking status**					**0.001**
Current smoker	183	73.2	253	83.5	
Ex-smoker	30	12.0	13	4.3	
Non-smoker	16	6.4	10	3.3	
Missing	20	8.0	27	8.9	
**Alcohol use**					**<0.001**
Current alcohol use	60	24.0	57	18.8	
Prior alcohol use	40	16.0	12	4.0	
No alcohol use	111	44.4	165	54.5	
Missing	38	15.2	68	22.4	
**Comorbidities**					
Psychiatric disorders	124	49.6	159	52.5	0.501
Hypertension	44	17.6	46	15.2	0.443
Type 2 diabetes	17	6.8	22	7.3	0.833
Coronary artery disease	15	6.0	8	2.6	0.049
Chronic lung disease	18	7.2	25	8.3	0.646
Hyperlipidemia	11	4.4	8	2.6	0.258
Acute kidney injury	17	6.8	32	10.6	0.121
Chronic kidney disease	14	5.6	9	3.0	0.123
Stroke	17	6.8	15	5.0	0.354
Peripheral vascular disease	16	6.4	6	2.0	0.008
Metastatic infections	8	3.2	22	7.3	0.036
**Psychiatric disorders**					
Substance use disorder	107	42.8	118	38.9	0.358
Depression	44	17.6	93	30.7	**<0.001**
Anxiety	50	20.0	74	24.4	0.215
Bipolar disorder	9	3.6	31	10.2	**0.003**
Post traumatic stress disorder	12	4.8	24	7.9	0.139
**Number of psychiatric disorders**					**0.039**
1	60	24.0	52	17.2	
2	33	13.2	44	14.5	
3 or more	31	12.4	61	20.1	
0 or missing	126	50.4	146	48.2	
**Valve**					
Tricuspid	112	44.8	199	65.7	**<0.001**
Mitral	64	25.6	72	23.8	0.617
Aortic	74	29.6	37	12.2	**<0.001**
Pulmonic	9	3.6	12	4.0	0.825
**Discharge status**					
Discharge alive	192	76.8	211	69.6	0.059
Death	23	9.2	17	5.6	0.105
Against medical advice	35	14.0	75	24.8	**0.002**
**Age**	**Median**	IQR**	**Median**	IQR**	**<0.001**
	35.66	13.07	32.19	13.28	

IE*: infective endocarditis; IQR**: inter-quartile range.

## Data Availability

Data underlying this study are from an approved repository that houses clinical data from the four healthcare systems in West Virginia. These data contain full Protected Health Information (PHI) and thus, legally cannot be shared publicly.

## References

[1] Ternhag A., Cederström A., Törner A., Westling K. (2013). A nationwide cohort study of mortality risk and long-term prognosis in infective endocarditis in Sweden. PLoS ONE.

[2] Duval X., Delahaye F., Alla F., Tattevin P., Obadia J.F., Le Moing V., Doco-Lecompte T., Celard M., Poyart C., Strady C. (2012). AEPEI Study Group. Temporal trends in infective endocarditis in the context of prophylaxis guideline modifications: Three successive population-based surveys. J. Am. Coll. Cardiol..

[3] Kiefer T., Park L., Tribouilloy C., Cortes C., Casillo R., Chu V., Delahaye F., Durante-Mangoni E., Edathodu J., Falces C. (2011). Association between valvular surgery and mortality among patients with infective endocarditis complicated by heart failure. J. Am. Med. Assoc..

[4] Iung B., Duval X. (2019). Infective endocarditis: Innovations in the management of an old disease. Nat. Rev. Cardiol..

[5] Pant S., Patel N.J., Deshmukh A., Golwala H., Patel N., Badheka A., Hirsch G.A., Mehta J.L. (2015). Trends in infective endocarditis incidence, microbiology, and valve replacement in the United States from 2000 to 2011. J. Am. Coll. Cardiol..

[6] Talha K.M., Dayer M.J., Thornhill M.H., Tariq W., Arshad V., Tleyjeh I.M., Bailey K.R., Palraj R., Anavekar N.S., Rizwan Sohail M. (2021). Temporal Trends of Infective Endocarditis in North America from 2000 to 2017-A Systematic Review. Open Forum Infect Dis..

[7] vom Steeg L.G., Klein S.L. (2016). SeXX Matters in Infectious Disease Pathogenesis. PLoS Pathog..

[8] Klein S.L., Flanagan K.L. (2016). Sex differences in immune responses. Nat. Rev. Immunol..

[9] Ingersoll M.A. (2017). Sex differences shape the response to infectious diseases. PLoS Pathog..

[10] Dohmen P.M., Binner C., Mende M., Daviewala P., Etz C.D., Borger M.A., Misfeld M., Eifert S., Mohr F.W. (2016). Gender-based long-term surgical outcome in patients with active infective aortic valve endocarditis. Med. Sci. Monitor..

[11] Curlier E., Hoen B., Alla F., Selton-Suty C., Schubel L., Doco-Lecompte T., Minary L., Erpelding M.-L., Duval X., Chirouze C. (2014). Relationships between sex, early valve surgery and mortality in patients with left-sided infective endocarditis analysed in a population-based cohort study. Heart.

[12] Murdoch D.R., Corey G.R., Hoen B., Miró J.M., Fowler V.G., Bayer A.S., Karchmer A.W., Olaison L., Pappas P.A., Moreillon P. (2009). Clinical presentation, etiology, and outcome of infective endocarditis in the 21st century: The International Collaboration on Endocarditis-Prospective Cohort Study. Arch. Intern. Med..

[13] Aksoy O., Meyer L.T., Cabell C.H., Kourany W.M., Pappas P.A., Sexton D.J. (2007). Gender differences in infective endocarditis: Pre- and co-morbid conditions lead to different management and outcomes in female patients. Scand. J. Infect. Dis..

[14] Polishchuk I., Stavi V., Awesat J., Golan Y.B.B., Bartal C., Sagy I., Jotkowitz A., Barski L. (2021). Sex differences in infective endocarditis. Am. J. Med. Sci..

[15] Elamragy A.A., Meshaal M.S., El-Kholy A.A., Rizk H.H. (2020). Gender differences in clinical features and complications of infective endocarditis: 11-year experience of a single institute in Egypt. Egypt. Heart J..

[16] Sevilla T., Revilla A., López J., Vilacosta I., Sarriá C., Gómez I., García H., San Román J.A. (2010). Influence of sex on left-sided infective endocarditis. Rev. Española Cardiol..

[17] Weber C., Gassa A., Rokohl A., Sabashnikov A., Deppe A.C., Eghbalzadeh K., Merkle J., Hamacher S., Liakopoulos O.J., Wahlers T. (2019). Severity of presentation, not sex, increases risk of surgery for infective endocarditis. Ann. Thorac. Surg..

[18] Bakir S., Mori T., Durand J., Chen Y.F., Thompson J.A., Oparil S. (2000). Estrogen-induced vasoprotection is estrogen receptor dependent: Evidence from the balloon-injured rat carotid artery model. Circulation.

[19] Zellweger R., Wichmann M.W., Ayala A., Stein S., DeMaso C.M., Chaudry I.H. (1997). Females in proestrus state maintain splenic immune functions and tolerate sepsis better than males. Crit. Care Med..

[20] Garcia M., Mulvagh S.L., Merz C.N., Buring J.E., Manson J.E. (2016). Cardiovascular Disease in Women: Clinical Perspectives. Circ. Res..

[21] Harris P.A., Taylor R., Thielke R., Payne J., Gonzalez N., Conde J.G. (2009). Research electronic data capture (REDCap)—A metadata-driven methodology and workflow process for providing translational research informatics support. J. Biomed. Inform..

[22] Khan M.Z. (2020). Racial and gender trends in infective endocarditis related deaths in United States (2004–2017). Am. J. Cardiol..

[23] Baddour L.M., Shafiyi A., Lahr B.D., Anavekar N.S., Steckelberg J.M., Wilson W.R., Sohail M.R., DeSimone D.C. (2021). A contemporary population-based profile of infective endocarditis using the expanded Rochester Epidemiology Project. Mayo Clin. Proc..

[24] Mori M., Brown K.J., Bin Mahmood S.U., Geirsson A., Mangi A.A. (2020). Trends in infective endocarditis hospitalizations, characteristics, and valve operations in patients with opioid use disorders in the United States: 2005–2014. J. Am. Heart Assoc..

[25] Nenninger E.K., Carwile J.L., Ahrens K.A., Armstrong B., Thakarar K. (2020). Rural–urban differences in hospitalizations for opioid use–associated infective endocarditis in the United States, 2003–2016. Open Forum Infect. Dis..

[26] Capizzi J., Leahy J., Wheelock H., Garcia J., Strnad L., Sikka M., Englander H., Thomas A., Korthuis P.T., Menza T.W. (2020). Population-based trends in hospitalizations due to injection drug use related serious bacterial infections, Oregon, 2008 to 2018. PLoS ONE.

[27] (2018). United States Census Bureau. ACS Demographic and Housing Estimates. https://data.census.gov/cedsci/table?g=0400000US54&y=2018.

[28] Slipczuk L., Codolosa J.N., Davila C.D., Romero-Corral A., Yun J., Pressman G.S., Figueredo V.M. (2013). Infective endocarditis epidemiology over five decades: A systematic review. PLoS ONE.

[29] DeSimone D.C., Lahr B.D., Anavekar N.S., Sohail M.R., Tleyjeh I.M., Wilson W.R., Baddour L.M. (2021). Temporal trends of infective endocarditis in Olmsted County, Minnesota, between 1970 and 2018: A population-based analysis. Open Forum Infect. Dis..

[30] Wurcel A.G., Anderson J.E., Chui K.K., Skinner S., Knox T.A., Snydman D.R., Stopka T.J. (2016). Increasing infectious endocarditis admissions among young people who inject drugs. Open Forum Infect. Dis..

[31] National Survey on Drug Use and Health (NSDUH), Substance Abuse and Mental Health Services Administration, Center for Behavioral Health Statistics and Quality. https://www.cdc.gov/nchs/data/hus/2019/020-508.pdf.

[32] Pazdernik M., Wohlfahrt P., Kautzner J., Kettner J., Sochman J., Stasek J., Solar M., Pelouch R., Vojacek J. (2019). Clinical predictors of complications in patients with left-sided infective endocarditis: A retrospective study of 206 episodes. Bratisl. Med. J..

[33] WV Department of Health and Human Resources, Health Statistics Center West Virginia Behavioral Risk Factor Surveillance System Report, 2018. http://www.wvdhhr.org/bph/hsc/pubs/brfss/2018/BRFSS2018.pdf.

[34] Galal H., Rifaei O., Abdel Rahman M., El-Sayed H. (2018). Prevalence and characteristics of tricuspid valve endocarditis among patients presented to Ain Shams Hospital echocardiography lab; one year study. Egypt. Heart J..

[35] Sambola A., Fernández-Hidalgo N., Almirante B., Roca I., González-Alujas T., Serra B., Pahissa A., García-Dorado D., Tornos P. (2010). Sex differences in native-valve infective endocarditis in a single tertiary-care hospital. Am. J. Cardiol..

[36] Thuny F., Giorgi R., Habachi R., Ansaldi S., Le Dolley Y., Casalta J.P., Avierinos J.F., Riberi A., Renard S., Collart F. (2012). Excess mortality and morbidity in patients surviving infective endocarditis. Am. Heart J..

[37] Cervera C., Castañeda X., de la Maria C.G., del Rio A., Moreno A., Soy D., Pericas J.M., Falces C., Armero Y., Almela M. (2014). Hospital Clinic Endocarditis Study Group. Effect of vancomycin minimal inhibitory concentration on the outcome of methicillin-susceptible Staphylococcus aureus endocarditis. Clin. Infect. Dis..

[38] Holland D.J., Simos P.A., Yoon J., Sivabalan P., Ramnarain J., Runnegar N.J. (2020). Infective endocarditis: A contemporary study of microbiology, echocardiography and associated clinical outcomes at a major tertiary referral centre. Heart Lung Circ..

[39] Trifunovic D., Vujisic-Tesic B., Obrenovic-Kircanski B., Ivanovic B., Kalimanovska-Ostric D., Petrovic M., Boricic-Kostic M., Matic S., Stevanovic G., Marinkovic J. (2018). The relationship between causative microorganisms and cardiac lesions caused by infective endocarditis: New perspectives from the contemporary cohort of patients. J. Cardiol..

[40] Ti L., Ti L. (2015). Leaving the hospital against medical advice among people who use illicit drugs: A systematic review. Am. J. Public Health.

[41] Kumar N. (2019). Burden of 30-day readmissions associated with discharge against medical advice among inpatients in the United States. Am. J. Med..

[42] Rudasill S.E., Sanaiha Y., Mardock A.L., Khoury H., Xing H., Antonios J.W., McKinnell J.A., Benharash P. (2019). Clinical outcomes of infective endocarditis in injection drug users. J. Am. Coll. Cardiol..

[43] Kraut A., Fransoo R., Olafson K., Ramsey C.D., Yogendran M., Garland A. (2013). A population-based analysis of leaving the hospital against medical advice: Incidence and associated variables. BMC Health Serv. Res..

[44] Tawk R., Dutton M. (2015). Racial differences in length of stay for patients who leave against medical advice from, U.S. general hospitals. Int. J. Environ. Res. Public Health.

[45] Yong T.Y., Fok J.S., Hakendorf P., Ben-Tovim D., Thompson C.H., Li J.Y. (2013). Characteristics and outcomes of discharges against medical advice among hospitalised patients. J. Intern. Med..

[46] Ahtela E., Oksi J., Sipilä J., Rautava P., Kytö V. (2019). Occurrence of fatal infective endocarditis: A population-based study in Finland. BMC Infect. Dis..

[47] West Virginia Department of Health and Human Resources (WVDHHR), Bureau for Public Health, Division of Health Promotion and Chronic Disease Fast Facts: Statistics about the population of West Virginia, US Census 2018. https://dhhr.wv.gov/hpcd/data_reports/Pages/Fast-Facts.aspx.

